# PI3K signaling and miRNA expression during the response of quiescent human fibroblasts to distinct proliferative stimuli

**DOI:** 10.1186/gb-2006-7-5-r42

**Published:** 2006-05-31

**Authors:** Jian Gu, Vishwanath R Iyer

**Affiliations:** 1 Section of Molecular Genetics and Microbiology, Institute for Cellular and Molecular Biology, Center for Systems and Synthetic Biology, University of Texas at Austin, 1 University Station A4800, Austin, TX 78712-0159, USA

## Abstract

**Background:**

Serum treatment of quiescent human dermal fibroblasts induces proliferation, coupled with a complex physiological response that is indicative of their normal role in wound-healing. However, it is not known to what extent such complex transcriptional events are specific to a given cell type and signal, and how these global changes are coordinately regulated. We have profiled the global transcriptional program of human fibroblasts from two different tissue sources to distinct growth stimuli, and identified a striking conservation in their gene-expression signatures.

**Results:**

We found that the wound-healing program of gene expression was not specific to the response of dermal fibroblasts to serum but was regulated more broadly. However, there were specific differences among different stimuli with regard to signaling pathways that mediate these transcriptional programs. Our data suggest that the PI3-kinase pathway is differentially involved in mediating the responses of cells to serum as compared with individual peptide growth factors. Expression profiling indicated that *let7 *and other miRNAs with similar expression profiles may be involved in regulating the transcriptional program in response to proliferative signals.

**Conclusion:**

This study provides insights into how different stimuli use distinct as well as conserved signaling and regulatory mechanisms to mediate genome-wide transcriptional reprogramming during cell proliferation. Our results indicate that conservation of transcriptional programs and their regulation among different cell types may be much broader than previously appreciated.

## Abstract 2

Global transcriptional profiling of human fibroblasts from two different tissue sources reveals distinct as well as conserved responses to different growth stimuli.

## Background

The transition of mammalian cells from quiescence to proliferation and their re-entry into the cell cycle (the G0 to G1 transition) underlies diverse normal physiological processes, such as tissue regeneration, wound healing and lymphocyte activation, and it is also one of the hallmarks of cancer [1-3]. This transition is marked by activation of cell-surface receptors, intracellular signal transduction pathways and effector transcription factors, which in turn lead to altered programs of gene expression, driving cells into the proliferative state. The molecular mechanisms governing this transition are believed to be distinct from those of other cell cycle transitions such as the G1 to S transition, and are less well characterized [4]. One key question is, to what extent are the regulatory pathways and transcriptional programs of re-entry into the proliferative state shared by different types of cells responding to different mitogenic signals? What is the interplay of these proliferative responses with the innate cell and tissue-type characteristics of cells?

Fibroblasts have long been used as a simple model to study mammalian cell proliferation in culture. The global transcriptional program of quiescent human dermal fibroblasts stimulated to proliferate by serum treatment indicates that there is a complex physiological wound-healing response that is superimposed upon the expected proliferative response [5]. Fibroblasts derived from different anatomical sites display characteristic expression patterns reflective of their site of origin [6]. Interestingly, the genes affected during the proliferative response of cultured fibroblasts to serum are predictive of tumor prognosis and metastasis in different cancers [7], implying that a conserved core set of regulatory mechanisms underlies the transition to proliferation in diverse cell types. On the other hand, gene expression analysis of cultured human fibroblasts from skin, lymph node, synovium and tonsil revealed heterogeneity in their expression profiles [8].

Despite these studies, however, little is known about the differences between the response of fibroblasts to serum versus other individual growth factors (GFs), both in terms of global transcriptional programs and the signal transduction pathways that are affected by each stimulus. It is unclear, therefore, if the complex mixture of components present in serum is required to trigger the wound healing response observed in skin fibroblasts. It is also not known if other types of fibroblasts that do not have an obvious role in surface wound healing and are not typically exposed to serum in the body are nevertheless capable of carrying out a similar program of gene expression. The connection between the G0 to G1 proliferative response and the physiological wound healing response is also not clear. For example, *AP-1 *is involved in cell cycle progression but it also targets genes in fibroblasts important for wound healing [9].

We address some of these questions in this study by analyzing the genome-wide transcriptional reprogramming of fibroblasts derived from skin as well as lung, when they are stimulated to proliferate either by serum or purified growth factors. We also dissect the contribution of specific signaling pathways to these global responses using an inhibitor of the PI3-kinase (PI3K) pathway. Finally, we have begun to analyze the potential involvement of micro RNAs (miRNAs) that have recently been shown to be involved in gene regulation in cancer models, in regulating the transition of normal quiescent diploid cells into the proliferative state.

## Results

### Experimental strategy

**Figure 1:**
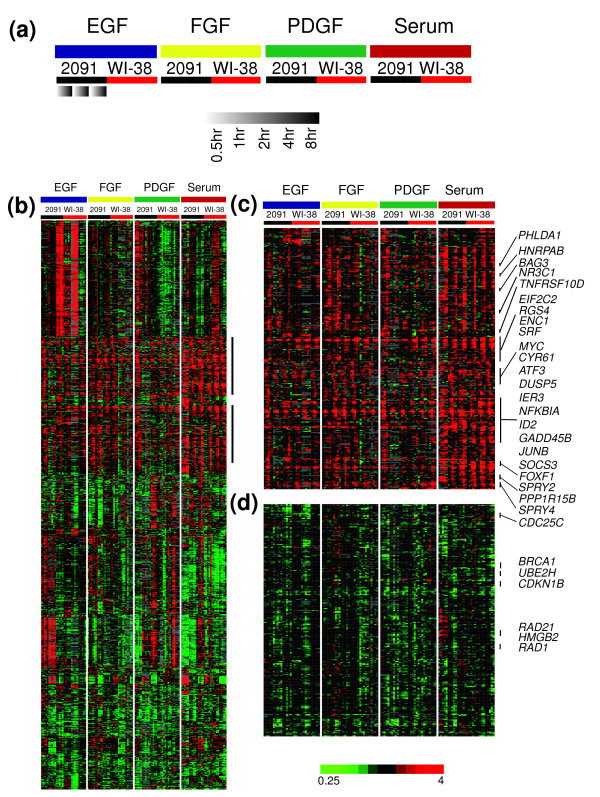
Experimental set up and overall expression profiles. **(a) **The time course of gene expression was determined during the response of two different fibroblast types; 2091 derived from foreskin and WI-38 derived from fetal lung. Each was treated with either the indicated GFs, epidermal growth factor (EGF), fibroblast growth factor (FGF), platelet derived growth factor (PDGF) or FBS (Serum), and three replicate time courses were run for each treatment. The layout of the samples in the other panels as well as in Figures 2-6 is as shown here. **(b) **Hierarchical cluster of 1,304 genes with a minimum expression change of twofold in at least 15 array experiments and with data present in at least 80% of all array samples. cDNAs with no known Unigene annotation or mapping to multiple Unigene clusters were removed. Black bars on the right indicate consistently induced genes. **(c) **Sub-cluster branches containing the consistently induced genes were selected and re-clustered. This set included 237 genes represented by 278 cDNA probes. **(d) **Consistently repressed genes were selected directly from expression data as described in Materials and methods and clustered. This set included 237 genes represented by 250 cDNA probes.

We profiled the response of two normal human diploid fibroblast cell lines to either serum or three different peptide GFs. One of the cell lines was a foreskin-derived dermal fibroblast (2091) and the other was a fetal lung-derived pulmonary fibroblast line (WI-38) [10]. Cells were first deprived of growth factors by growing them in medium containing 0.1% fetal bovine serum (FBS) for 48 hours, then treated with medium containing either 10% FBS, epidermal growth factor (EGF), fibroblast growth factor (FGF) or platelet derived growth factor (PDGF). Cells were harvested at 6 different time points (0 h, 0.5 h, 1 h, 2 h, 4 h, 8 h), followed by total RNA isolation and RNA amplification (Figure 1a). Temporal global transcription profiles were measured using cDNA microarrays containing 46,544 clones, corresponding to approximately 31,158 unique Unigene clusters [11,12]. RNA from each sample of cells was reverse transcribed to cDNA and labeled with Cy5, and hybridized to the cDNA microarrays together with a universal human reference sample labeled with Cy3.

To identify consistent expression changes caused by each stimulus, we performed three independent biological replicates of each treatment for each cell line (Figure 1a). In most cases, we isolated at least two independent time zero samples for each treatment time course and normalized all expression changes relative to the average of the time zero values to minimize error arising from time zero measurements. All data were uploaded to a relational database [13] and filtered on basic data quality measures before further analysis. All primary data reported here are available at NCBI's GEO (Accession IDs GSE3901 and GSE3902) as well as at the authors laboratory web site [14], and the ratio data tables for all gene sets described here are available as accompanying Additional data files. A small subset of the expression changes we observed were not reproducible across all three repeats for each treatment. To ensure that we analyzed only data that was biologically reproducible, the results and discussion presented here pertain to only those changes that were consistent across the three biological replicate time-courses of a given combination of cell line and treatment. Three classes of genes will be discussed in the following sections. Class I refers to genes that were consistently induced or repressed across all the serum and GF treatments. Class II refers to genes that were differentially expressed in response to serum and the GF treatments. Class III genes are those that were differentially expressed between the two fibroblast cell lines.

### The expression of wound healing genes is affected by all treatments and is not specific to serum

We used hierarchical cluster analysis to obtain a bird's-eye view of the global expression patterns (Figure 1b). Strikingly, there were no prominent sets of genes that were consistently and uniquely regulated by a given treatment. However, genes showing statistically significant differences in expression between serum treatment and the three other GF treatments could be identified through the use of a *t* test (Class II). These treatment-specific responses are described in a later section, as are the genes that show differences in response between the two cell types (Class III). We identified a group of 237 genes, represented by 278 cDNA probes, that were concordantly induced by serum and each of the three different growth factors (Figure 1c). To identify genes consistently repressed by all four treatments, we selected genes whose expression was lower after stimulation than in the time zero samples in at least 85% of arrays. This identified a set of 237 genes represented by 250 cDNA probes (Figure 1d). Together, this set of genes, which generally showed similar responses in both cell types to all four treatments, is referred to as Class I (Additional data file 1).

**Figure 2:**
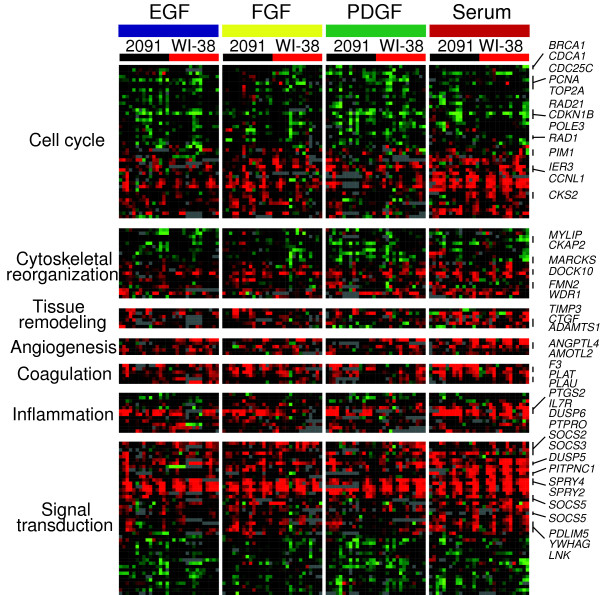
Functional groupings in consistently expressed genes. Genes with consistent expression patterns across all treatments (Class I genes) were manually grouped into several functional categories as indicated, based on annotations from Unigene and GO.

Class I genes included known immediate-early genes such as *JUNB*, *MYC*, *PTGS2 *and others. It has been previously noted that in response to serum, fibroblasts show altered expression of genes involved in cell proliferation, blood coagulation, cytoskeletal reorganization, angiogenesis and inflammation - all functions that are closely related to the physiology of wound healing [5,7]. Surprisingly, our results indicate that not only serum, but several other growth factors in isolation affect the expression of a similar coherent set of genes relevant to wound healing. We observed the altered expression of several genes involved in cytoskeletal reorganization, tissue remodeling, angiogenesis, blood coagulation and inflammation, as well as signal transduction pathways that mediate the above processes (Figure 2). We used DAVID [15,16] to quantify the enrichment of Gene Ontology (GO) terms in our gene sets and noted significant enrichment of several of these categories (Additional data file 2). Moreover, the response of lung fibroblasts with regard to the expression of these genes could not be distinguished from the response of the skin fibroblasts, indicating that the wound healing expression signature is not specific to the response of dermal fibroblasts to serum.

### The core conserved response of fibroblasts during the transition from quiescence to proliferation

The molecular pathways underlying the transition from quiescence to proliferation triggered by each of the four treatments in both fibroblast types appeared to be strongly conserved, as suggested by the altered expression of cyclin/cdk related genes, such as *CCNL1*, *CKS2*, *CDCA1*, *CDKN1B *and *CDC25C*, and genes involved in DNA replication, such as *TOP2A*, *PCNA *and *POLE3*, across all treatments (Figure 2). Interestingly, a group of genes related to cell cycle checkpoints, such as *BRCA1*, *RAD1 *and *RAD21*, were repressed (Figure 2), likely reflecting the requirement for the down-regulation or deactivation of these checkpoint proteins for either cell cycle entry or progression. Genes with anti-apoptotic function, such as *BAG3 *and *TNFRSF10D*, were upregulated while others promoting apoptosis were inhibited in all the treatments (Figure 1c). *GADD45B*, another induced gene, is believed to have anti-apoptotic functions by downregulating JNK signaling [17]. All these observations suggest that reentry into the cell cycle by serum starved G0 cells requires the upregulation of cell cycle related genes as well as inhibition of apoptotic signaling pathways.

**Figure 3:**
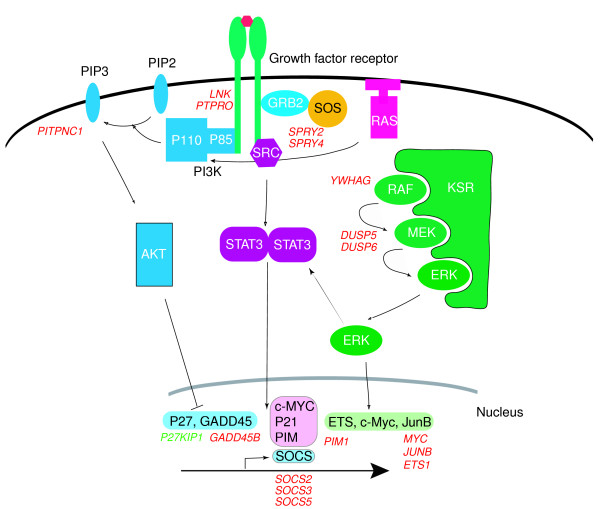
Signaling pathways activated by serum and GF treatments. Major components from three pathways, the PI3K pathway, the JAK/STAT pathway and the MAP kinase pathway are indicated. Class I genes with consistent expression profiles across all treatments in our experiments are indicated by red (up-regulated) or green (down-regulated) at their appropriate position in each of the pathways.

Cell cycle progression from G0 to G1 is highly dependent on the activation of intracellular signaling pathways. Growth factors are required at two critical points during the G0 to G1 transition to stimulate the MAP kinase (ERK) pathway, *c-Myc*, and the PI3K pathway [18]. We observed altered expression of regulatory factors in the RAS-MAPK pathway and the PI3K pathway, such as *YWHAG *(14-3-3- gamma) [19], *SPRY2 *[20], *DUSP5 *[21], *DUSP6 *[22], and *PITPNC1 *[23] (Figure 2). This observation suggests that both signaling pathways are involved in the response of fibroblasts but, interestingly, only the MAPK pathway activation and *c-Myc *induction are believed to be indispensable during the early 0 to 8 hour period of fibroblast response [18]. Our results also corroborate previous studies showing that different growth factors may activate cell proliferation by largely overlapping mechanisms that include the activation of these two signaling pathways [24]. The potential involvement of the JAK/STAT pathway was indicated by the upregulation of its downstream effectors, such as *PIM1 *[25], as well as the upregulation of several *SOCS *genes, which are targets and negative feedback regulators of this pathway [26]. Figure 3 shows the altered expression of these genes overlaid on a schematic representation of the three signaling pathways whose activity is suggested by this expression profiling data.

There were subtle differences among the induction patterns of Class I genes even though they were consistently induced by all four treatments. Although serum treatment generally resulted in higher peak induction levels than the growth factor treatments, the majority of the induced genes peaked eight hours after serum addition compared to 2 to 4 hours after GF treatment (Additional data file 3). This could partially be a result of differences in concentrations of the GFs that were employed. Indeed, we found that increasing concentrations of FGF, ranging from 5 ng/ml to 135 ng/ml, caused increasingly stronger induction of these genes at two hours (Additional data file 4). However, the fact that serum generally caused stronger induction of genes but with delayed kinetics suggests that there are differences in the response of fibroblasts to serum and the GFs such as those described below or, possibly, that a combination of different growth factors and other components such as lysophosphatidic acid in serum could contribute to the differences.

### The PI3-kinase pathway is differentially involved in regulating the responses to serum versus individual growth factors

Since hierarchical clustering did not directly reveal genes with expression differences specific to each treatment, we used a supervised approach to identify genes whose average expression levels were significantly different after treatment among the different groups. Based on the fact that peak expression levels were most different between serum treatment and all the growth factor treatments, we used a *t* test to identify genes that showed significant differential expression between these two groups. This analysis identified 701 cDNAs, representing 619 genes, that tended to be induced following serum treatment but were repressed or remained unchanged after GF treatment, and 613 cDNAs, representing 566 genes, that generally showed higher expression levels in response to growth factors. These genes are termed Class II genes and are described below (Additional data file 5).

**Figure 4:**
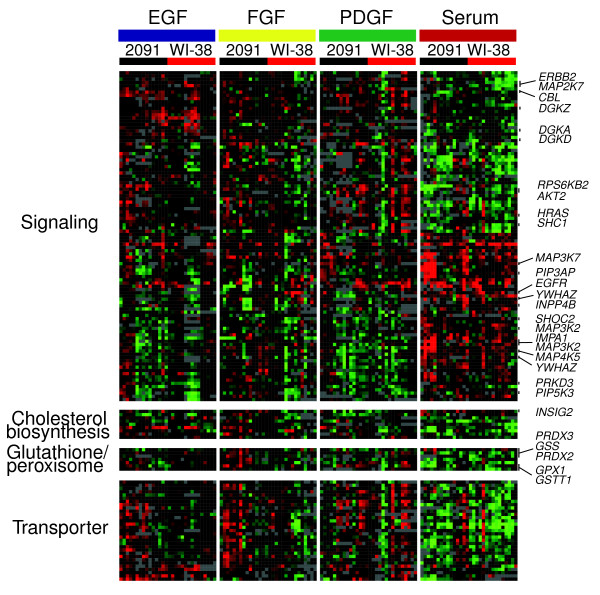
Class II genes showing differential expression responses to serum versus GFs. Genes in this category were identified through the use of a *t *test with false discovery rate (FDR) less than 1%. The two groups for the *t *test were all the serum treated samples as one group, and all the GF treated samples as another. Genes involved in signal transduction, cholesterol biosynthesis, glutathione/peroxisome synthesis and transporters are indicated.

**Figure 5:**
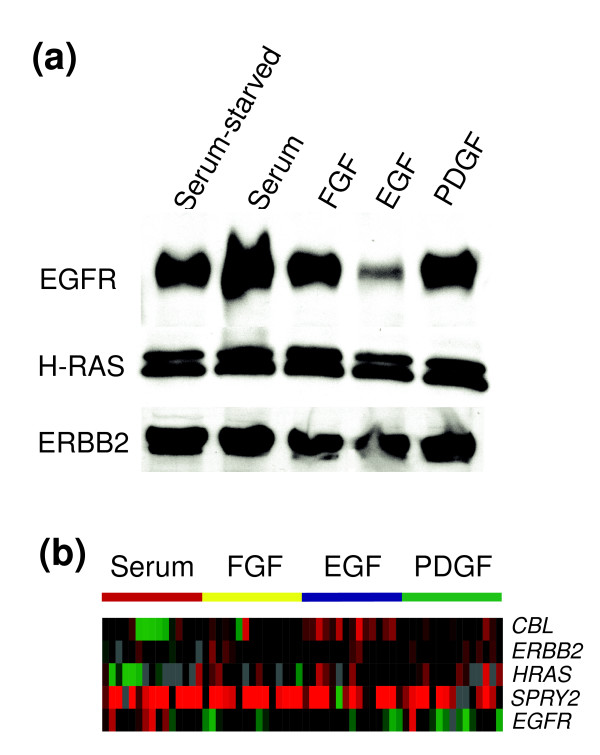
A putative regulatory circuit involving *EGFR*. **(a) **Protein expression levels in skin fibroblasts 2 hours after treatment with serum or individual growth factors. Total protein extracts from treated cells were loaded equally on a gel followed by western blot analysis using *EGFR*, *HRAS *and *ERBB2 *antibodies. **(b) **mRNA expression patterns for *CBL*, *ERBB2*, *HRAS*, *SPRY2 *and *EGFR *across all growth stimulations in foreskin fibroblasts. *EGFR *mRNA is more strongly induced by serum compared to the GFs, but the induction of EGFR protein is only modest in response to serum. Its negative regulator, CBL, is more strongly induced at the RNA level in response to EGF, concordant with the strong down-regulation of EGFR protein in EGF treated cells.

Many Class II genes were those involved in signal transduction, suggestive of differences in signaling events between the serum and GF responses. The EGF receptor gene *EGFR *was induced by serum, while its negative regulator *CBL *- an E3-ubiquitin ligase that targets *EGFR *and *FGFR *for degradation [27] - was upregulated in the GF treatments (Figure 4), suggesting a negative regulatory circuit. We observed a modest increase in *EGFR *protein levels in response to serum compared to the GF treatments (Figure 5a). However, EGF treatment, but not PDGF or FGF, caused a marked down regulation of *EGFR *protein levels, even though at the transcriptional level the response of *EGFR *to the three different growth factors was consistently similar. This is likely due to a stronger induction of *CBL *by EGF compared to the other GF treatments (Figure 5b), suggesting that the negative regulatory circuit involving *CBL *and *EGFR *is involved in mediating the response to growth signals in these cells. Although we could validate the expression levels and potential regulation of *EGFR *at the protein level, there were cases where protein levels did not reflect the changes in mRNA expression levels. For example, *H-RAS *transcript levels were slightly induced in GF treatments compared to serum. However, we failed to detect any change in *H-RAS *protein expression levels (Figure 5a,b).

The PI3K pathway is believed to be involved during late G1 during the transition from quiescence to proliferation [18]. Some downstream components of PI3K signaling, such as *PIP5K3*, were among the Class II genes that were induced by serum, while others like ribosomal protein S6 kinase B6 and *AKT2 *[28-30] were among the serum repressed Class II genes (Figure 4). Diacylglycerol (DAG) produced by the activation of the phosphoinositide pathway is the physiological activator of protein kinase C (PKC) as well as other protein kinase C conserved region 1 (C1)-domain proteins, such as protein kinase D1 (PKD1), RasGRPs and DAG kinase gamma [31]. Diacylglycerol kinases (DGKs) terminate DAG-mediated signaling by converting DAG to phosphatidic acid (PA). Nine DGK isoforms have been identified and classified into five subgroups based on their structure, which, along with their different subcellular localization, suggests distinct DAG signaling events they may regulate [32]. *DGKs*, such as *DGKA*, *DGKD*, and *DGKZ*, were among the serum repressed Class II genes and *PKC *(*PRKD3*) was in the serum upregulated set. Taken together, these observations suggested that the PI3K signaling pathway may have a more prominent role in the response to serum (Figure 4).

**Figure 6:**
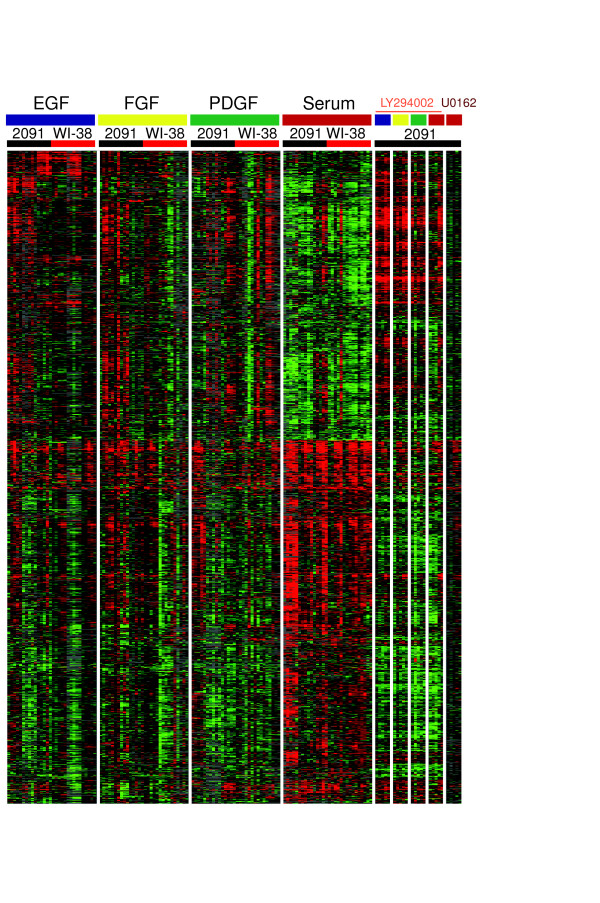
Role of the PI3K pathway in mediating differences between serum and GF response. A cluster view of the 1,379 Class II clones showing differential expression between serum and other GF treatment groups is shown. The right hand side shows the expression profiles of these Class II genes in foreskin fibroblasts when quiescent cells were first treated with either LY294002 (a PI3K inhibitor) or U0126 (a MAPK pathway inhibitor) before growth stimulation with serum or growth factors for LY294002 or serum for U0126. Inhibition of the PI3K pathway, but not the MAPK pathway, converted the expression profiles of Class II genes after serum treatment to a pattern similar to that after GF treatment.

To test the hypothesis that the PI3K pathway is responsible for some of the differences in the response of fibroblasts to serum versus individual GFs, we treated cells with the PI3K pathway inhibitor LY294002 and determined expression profiles after growth stimulation. When the PI3K pathway was inhibited, the response of Class II genes in the serum treatment group switched to a pattern similar to that of the GF groups, consistent with the notion that the PI3K signaling pathway has a more prominent role in the response to serum (Figure 6). This switch of Class II expression profiles from serum-like to GF-like was specific to inhibition of the PI3K pathway. When cells were treated with U0126 - a MEK inhibitor - prior to serum stimulation, we did not observe a similar switch in expression profiles (Figure 6). However, the serum response was almost completely abrogated after U0126 treatment, consistent with a critical role for the MAP kinase pathway in cell cycle reentry.

### Identification of genes differentially expressed between cell lines

**Figure 7:**
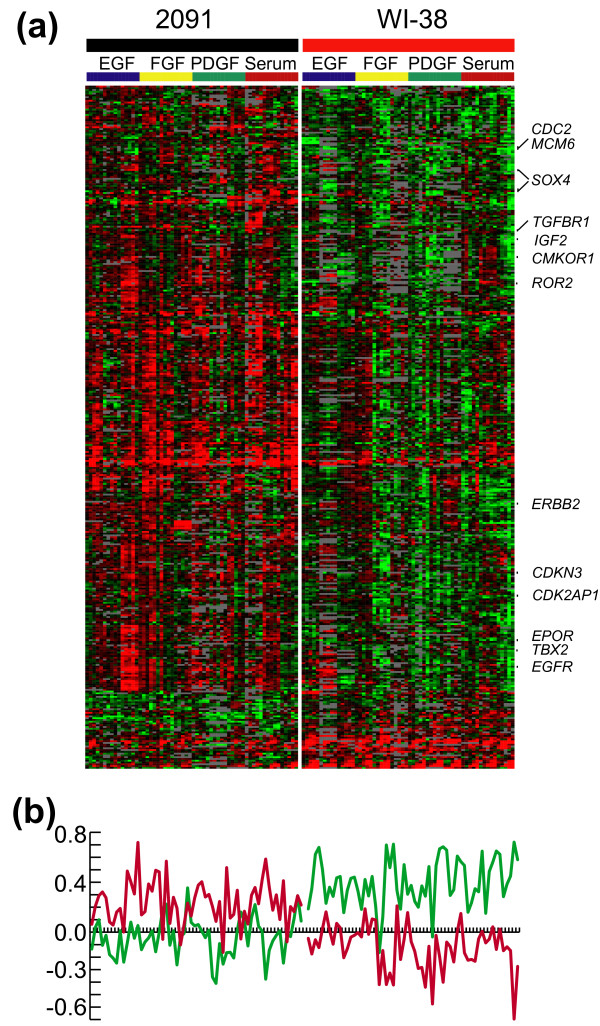
Class III genes differentially expressed between skin and lung fibroblasts during their transition from quiescence to proliferation. **(a) **A cluster of 385 genes differentially expressed between skin and lung fibroblasts, identified by using a *t *test and setting the FDR to 1%. **(b) **Average profiles of genes that were either highly expressed (red line) or repressed (green line) in skin fibroblasts relative to lung fibroblasts. The majority of Class III genes are in the former category and many of them reflect the higher proliferation rate of the skin fibroblasts.

Fibroblasts from different anatomical sites tend to have characteristic expression patterns related to their specific physiological functions despite sharing similar morphology [6]. The two fibroblast cell lines we used originate from different tissue sources - skin and lung - and are thus expected to have distinct transcription programs. During quiescence, namely, at the zero-hour time point and in the absence of growth signals, lung-specific genes were expressed in the WI-38 cell line derived from fetal lung whereas skin-specific genes were expressed in the 2091 cell line derived from newborn foreskin (Additional data file 6). To identify differences between the responses of the two cell types to growth stimulation, however, we compared the relative expression ratios after stimulation from all the experiments on the two cell lines using the same method as described above. A set of 385 cDNA probes representing 358 genes was found to be differentially expressed between these two cell lines at a false discovery rate (FDR) of 1% (Figure 7). This set of genes is denoted as Class III (Additional data file 7).

Hierarchical clustering of the 385 Class III genes revealed two broad patterns of differential expression between the two cell types; 341 genes were generally more strongly induced in the skin fibroblasts whereas only 44 genes were expressed at higher levels in the lung fibroblasts (Figure 7a,b). This bias is possibly due to the higher proliferative potential of the foreskin cells. Several genes related to cell proliferation, such as *CDC2*, *CDK2AP1*, *CDKN3 *and *MCM6*, tended to be more strongly induced in the skin cells. Several genes encoding cellular receptors, such as erythropoietin receptor, GABA A receptor, receptor tyrosine kinase-like orphan receptor 2, chemokine orphan receptor 1, EGF and the ERBB2 receptor, were included among the genes more strongly induced in the skin fibroblasts relative to the lung fibroblasts (Figure 7a). Only a few previously characterized skin-specific genes were included among the genes more strongly induced in the skin cells. One example of such a gene is *TBX2*, which remained unchanged or showed slightly reduced expression in most treatments of the lung cells. Interestingly, however, a few lung signature genes, such as *TBX2 *and *SOX4*, were among the genes induced to a greater extent in the foreskin cell line (Figure 7a). Since these genes are expressed at much higher levels in lung than in foreskin, it is possible that their induction is more easily detectable in the skin cells. Most lung and skin specific genes did not show significant expression changes during the transition from quiescence to proliferation, and it is likely that their expression levels remain constant during the cell cycle.

### miRNA profiling of skin fibroblasts in response to growth stimulation

We noticed that the set of Class I genes consistently induced by all treatments included *EIF2C2*, a member of the Argonaute gene family and a component of the RISC complex involved in post-transcriptional gene silencing by miRNAs [33,34] (Figure 1c). Although miRNAs have recently been shown to have a role in the proliferation of cancer cells and stem cells [35-37], not much is known about their role in the proliferative response of normal, differentiated quiescent cells. We therefore performed expression profiling with miRNA microarrays to explore the alterations in the expression levels of miRNAs in skin fibroblasts during their transition from quiescence to proliferation. miRNA was isolated from asynchronously growing cells and from quiescent fibroblasts, before and after growth stimulation. We carried out a total of 18 miRNA microarray hybridizations using miRNA from six independent biological samples and dye-swap hybridizations. The overall expression changes in miRNAs were less dramatic compared to their expression differences reported in different human tissues [38]. We also noted a higher degree of experimental variability within biological repeats and dye-swap experiments, possibly due to the labeling method we employed, which relies on adding dye modified nucleotides directly to the miRNAs. Dye quenching can occur at sub-optimal densities of labeling, which results in fluorescent dyes in close proximity to one another [39,40].

**Figure 8:**
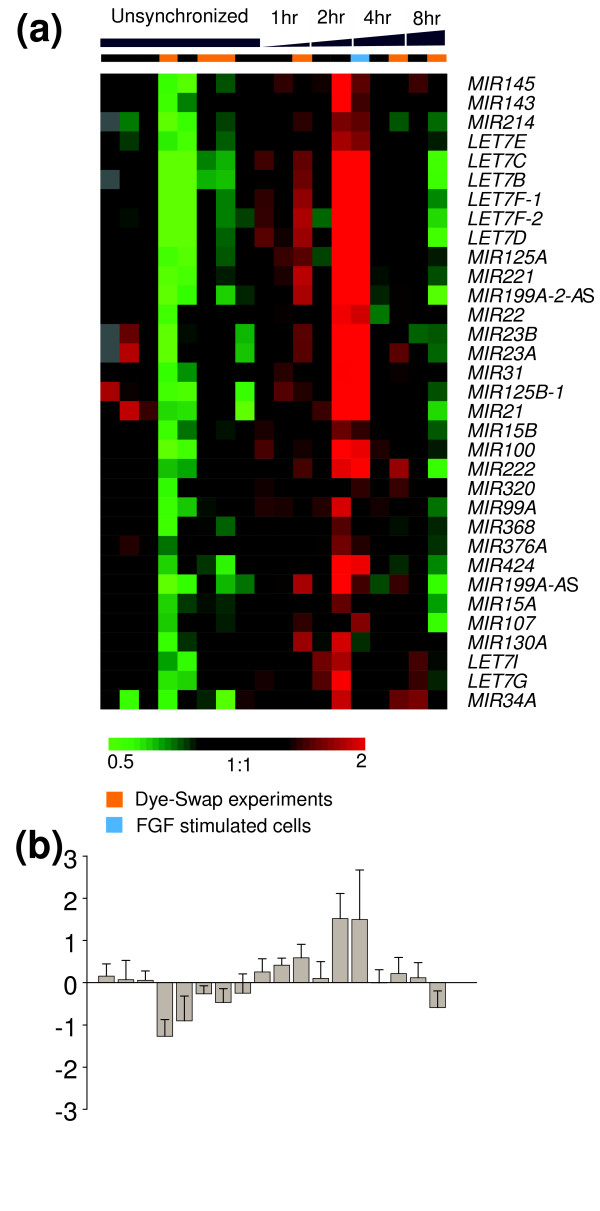
Expression profiles of miRNAs in skin fibroblasts. **(a) **A cluster of 33 miRNAs induced during early proliferation but repressed in asynchronously growing cells and late during proliferation. 10% serum was used in all these stimulation treatments except for one FGF (100 ng/ml) treatment as indicated. The colors representing ratios from the dye-swap experiments were inverted to be consistent with the non-dye swap samples. **(b) **Averaged expression level for each condition in the above clustering image.

We identified a cluster of 33 miRNAs with similar and consistent expression profiles across the replicates and dye-swaps. This cluster of miRNAs was repressed in asynchronously growing skin fibroblasts but they were induced early during proliferation, both by serum as well as FGF. This cluster includes a number of miRNAs belonging to the *let-7 *family as well as several other miRNAs (Figure 8, Additional data file 8), suggesting that these miRNAs might be involved in regulating the expression of target genes important for the reentry of these cells into the cell cycle.

We used the PicTar program [41,42] to predict miRNA targets based on sequence homology, optimal free energy, and ortholog searching [41]; 31 of the 33 miRNAs were found in the PicTar database. Predicted targets for these miRNAs with a PicTar score greater than 4 comprised 1,246 unique Unigene clusters. Functional annotation analysis using DAVID revealed that genes involved in the MAP kinase pathway, focal adhesion and GAP junctions were among the most enriched Kegg pathways (*p *< 0.01). However, a similar analysis of targets for 31 random miRNAs also revealed an apparent enrichment of MAP kinase pathway genes, so the biological meaning of the enrichment of these categories in the PicTar predicted targets of the serum induced miRNAs remains unclear.

## Discussion

### The wound healing and cell proliferation response of human fibroblasts

The characteristic wound healing and proliferative response of human dermal fibroblasts after serum treatment originally suggested that this response reflected the obligatory *in vivo *physiological response of dermal fibroblasts to serum factors released upon wounding. Here we observe that not only dermal fibroblasts, but also lung fibroblasts, carry out a largely conserved program of gene expression reminiscent of wound healing, in response not only to serum but also individual purified GFs. Although some aspects of this conserved response could arise due to the similarity of culture conditions, the fact that tissue-specific differences were maintained in quiescent fibroblasts even in culture suggests that the wound healing response to ostensibly proliferative stimuli is more broadly conserved across distinct fibroblast cell types from different tissue sources and can be elicited by a variety of triggers. Conceivably, the wound healing response originated initially in a dermal-like fibroblast and persisted in other fibroblast types in other specialized tissues. Although it is possible to speculate on the evolutionary reasons and advantages of such a conserved gene expression program, the mechanisms of how the program is initiated and regulated are unclear. One possibility is that, in fibroblasts, the wound healing and cell signaling programs are coupled to a large extent to cell proliferation. In all the experiments in this study and in several previous studies, the signals that trigger the wound healing gene expression program also caused concomitant cell proliferation [43]. However, some agents such as phenytoin can induce wound healing genes, including those involved in tissue remodeling, inflammation, coagulation and hemostasis in dermal fibroblasts, without inducing cell proliferation, suggesting that the wound healing response does not necessarily require cell proliferation [44]. Mechanical strain in human scleral fibroblasts [45] can also induce similar functional groups of genes that are indicative of wound healing, although there were few individual genes commonly induced in both studies.

The effect of different stresses, such as heat shock, ER stress, oxidative stress and crowding stress, on lung fibroblasts have been examined, and it was observed that the response to endoplasmic reticulum (ER) stress caused by dithiothreitol (DTT) was to some extent the opposite of the serum response [46]. Indeed, we noted that some cell cycle control genes showed differences between our experiments and the DTT treatments. For example, p27 Kip1 (*CDKN1B*) was repressed in our experiments in response to serum and GFs, but induced by DTT treatment, while *CKS2 *and *GSPT1 *showed the opposite behavior. At the same time, however, ER stress induced several common immediate early response (IER) genes, such as *Jun-B*, *IER3*, *SNAIL*, *TNFAIP3*, *GADD45B*, and *SPRY2*, in the same manner as serum and GFs in the experiments reported here, suggesting that the expression of the these IERs are involved not only in cell proliferation in fibroblasts, but also cell survival in response to DTT induced stress.

### Signaling pathways mediating transcriptional programs in fibroblasts

The concordant up or down regulation of a large set of genes by distinct GF treatments as well as serum (Figure 1c,d) is likely due to the fact that many different growth factor receptors share a conserved intracellular receptor tyrosine kinase (RTK) domain, which triggers similar downstream events upon ligand (GF) binding. Global expression profiling studies with chimeric receptor derivatives in mouse fibroblasts indicate that the different intracellular signaling domains of a growth factor receptor, although they may activate distinct signal transduction pathways, induce largely overlapping sets of genes [24].

Despite the largely overlapping transcriptional response of the different mitogenic treatments, we could identify hundreds of genes that were differentially regulated when we compared serum treatment with the other growth factors (Class II genes, Figure 4). Since serum is a better cell proliferation stimulus than the other growth factors [47-49], we firstly expected to see differences in the expression of cell cycle related genes. Indeed, cyclin C and *CDK6*, which are believed to be especially important in the G0 to G1 transition of cells [4], are among the set of genes more strongly upregulated by serum (Figure 6). However, the actual expression differences of these two genes among the two groups are minor and it is not clear how significant they are. Secondly, one would expect to see differences in signaling pathways because serum would be expected to activate not only growth factor related RTKs, but also other cytokine RTKs or hormone related G-protein coupled receptors. Indeed, we observed differences in the expression of many genes involved in mediating the MAP kinase, PI3 kinase, DAG and G-coupled receptor pathways. Interestingly, treatment with a PI3K pathway inhibitor specifically reduced or eliminated most of these differences, suggesting that the PI3K pathway has a more prominent role in the response of fibroblasts to serum.

### Conserved and specialized gene expression programs and regulation

The fibroblasts we used originated from different tissue sources (skin and lung) and their slightly different transcription profiles in response to mitogenic stimulation may partly reflect their specialized physiological function in their tissue of origin. Anatomic origin-specific gene expression patterns for these cells have been described before [6], but the expression of most of those genes did not vary during the proliferative response in our study. Out of the 358 genes that were differentially expressed between the two cell types, around one-quarter of the annotated genes were related to cell cycle and signaling processes. The former group of genes reflects the fact that the proliferation rate of the WI-38 lung fibroblasts was considerably slower than that of the 2091 skin fibroblasts.

The presence of a cluster of miRNAs induced during early proliferation suggests that they may be involved in regulating this transition in quiescent fibroblasts. Some of the miRNAs in this cluster, such as *miR-125b*, *miR-143*, *let-7 *and *miR-21*, have recently been discovered to be related to cell differentiation, proliferation and apoptosis [50-53]. A role for *miR-21 *and *let-7 *in human fibroblast proliferation would be consistent with the finding that a conditional knockout of the miRNA processing enzyme dicer in mouse primary fibroblasts affects the expression of *miR-21 *and *let-7*, and slows down the growth rate of fibroblasts [54]. The expression pattern of these miRNAs in our study was well correlated with that of genes encoding immediate early transcription factors, such as *c-Myc*, *SRF*, *JUNB*, *EGR2 *and *EGR3*. Interestingly, *c-Myc *and *SRF *are known to regulate the expression of some miRNAs [55,56]. The correlation between the expression profiles of miRNAs and immediate-early transcription factors suggests possible co-regulation of miRNAs and other regulators by common immediate early transcription factors.

Our data support the existence of a conserved cell cycle gene progression program and, surprisingly, a conserved wound healing expression program among fibroblasts of distinct origins responding to distinct proliferative stimuli. Although tissue specific differences exist between these two types of cells, these specialized genes are, in general, not strongly responsive to growth stimuli. It will be interesting to similarly study commonalities and distinctions between growth-related expression programs among cells of more diverse origins, and dissect the contributions of individual regulatory proteins and non-coding RNAs to such global expression programs.

## Materials and methods

### Cell culture and growth factor/inhibitor treatments

Normal human diploid foreskin fibroblast cell line 2091 and lung fibroblasts WI-38 (ATCC, Manassas, VA, USA) at passage number 5 to 10 were used. Cells were cultured in Dulbecco's modified Eagle's medium (DMEM) supplemented with 10% FBS and 100 units penicillin-streptomycin. Unsynchronized cells were normally growing cells at 80% to 90% confluence. For serum starvation, cells were grown to about 60% confluence in 10 cm petri dishes and washed three times with DMEM without serum. Low serum (0.1% FBS) DMEM was then added to the dishes followed by a 48 h incubation. Then, the medium was replaced by fresh DMEM with 10% FBS or DMEM with 0.1% FBS plus different GFs. GFs (Upstate, Charlottesville, VA, USA) were prepared according to supplier's instructions and added to the medium to a final concentration of 50 ng/ml (EGF), 100 ng/ml (FGF) and 40 ng/ml (PDGF). For LY294002 treatment, serum starved 2091 cells were treated with 25 μM LY294002 (Promega, Madison, WI, USA) for 30 minutes prior to GF or serum treatment. Cells were collected right before LY294002 treatment, before serum/GF stimulation (0 h) and 5 subsequent intervals (0.5, 1, 2, 4 and 8 h). For U0126 treatment, serum starved 2091 cells were treated with 10 μM U0126 (Promega) for 15 minutes prior to 10% serum treatment. Cells were collected at the same time points as for LY294002.

### RNA isolation and amplification

Total RNA was isolated with Trizol reagent (Invitrogen, Carlsbad, CA, USA) according to the manufacturer's instructions. Total RNA quantity and quality was determined by a high-resolution electrophoresis system (Agilent Technologies, Palo Alto, CA, USA). Universal human reference RNA (Stratagene, La Jolla, CA, USA) was used for the reference channel in all hybridizations. RNA amplification was based on the methodology developed at Stanford University [57]; 1 μg total RNA was co-denatured with 0.5 μg T7-oligo dT primer (GCATTAGCGGCCGCGAAATTAATACGACTCACTATAGGGAGA-(dT)21V) at 70°C for 5 minutes and then chilled on ice. Reverse transcription was carried out in a 20 μl reaction, containing 1 μl of SuperScript II (200 U/μl; Invitrogen), 1 μl RNase inhibitor (40 U/μl; Promega), 4 μl of 5x 1st strand buffer, 2 μl of 0.1 M DTT, 1 μl diethylpyrocarbonate (DEPC) treated water, and 1 μl of 10 mM dNTP mix (PE Applied Biosystems, Foster City, CA, USA). After 2 hours incubation, 2nd strand cDNA synthesis was carried out by adding 2.5 μl *Escherichia coli *DNA polymerase I (10 U/μl; NEB, Beverly, MA, USA), 1.5 μl *E. coli *DNA ligase (10 U/μl; NEB), 1 μl RNase H (10 U/μl; Ambion, Austin, TX, USA), 3 μl of 10 mM dNTP mix, 10 μl of 10x 2nd strand synthesis buffer, and 62 μl DEPC-treated water. The reaction was incubated at 16°C for 2 hours, followed by DNA purification. 1 mg linear acrylamide (Ambion) and 100 μl Phenol:Chloroform:Isoamyl Alcohol (25:24:1; Invitrogen) were added to the reaction. The aqueous phase was then isolated using phase lock gel (Eppendorf, Hamburg, Germany) followed by the addition of 50 μl of 7.5 M ammonium acetate, and 375 μl of 100% ethanol. The cDNA was precipitated immediately by centrifugation and washed twice with 70% ethanol. The cDNA pellet was resuspended in 8 μl water and subject to *in vitro *transcription using the T7 MegaScript kit (Ambion) followed by purification using the RNeasy Mini kit (Qiagen, Hilden, Germany) according to manufacturer's instructions.

### microRNA preparation

Total RNA was isolated using either the mirVana miRNA Isolation Kit (Ambion) or Trizol (Invitrogen) according to manufacturers' instructions. A FlashPAGE fractionator (Ambion) was used to isolate miRNA from total RNA. Briefly, 75 μg total RNA sample was mixed with a dye and loaded onto a denaturing acrylamide gel matrix. RNAs smaller than 40 nucleotides were recovered after fractionation using the flashPAGE Reaction Cleanup kit. The mirVana miRNA labeling Kit (Ambion) was then used to label the enriched miRNAs. A mixture of unmodified and amine-modified nucleotides were added to the 3' end of each miRNA by poly(A) polymerase. The amine group on the modified miRNA was coupled to NHS-ester linked Cy5 or Cy3 dyes (GE Healthcare (formerly Amersham Bioscience), Piscataway, NJ, USA). Unincorporated dyes were removed with a glass fiber-based filter after a one hour coupling reaction.

### cDNA microarrays

We printed cDNA microarrays on poly L-lysine coated microscope slides using a custom-built robotic arrayer. Each slide was printed with about 47,000 sequence-verified IMAGE clones (Research Genetics/Invitrogen). Labeled cDNA from 3 μg of Amplified Universal Human Reference total RNA and amplified total RNA extracted from cells were used as the two channels for each hybridization. The amino-allyl labeling and hybridization protocols are described online [58]. Cy3 and Cy5 labeled cDNAs were mixed with 10 μg human Cot-1 DNA, 5 μg polyA RNA, and 5 μg yeast tRNA (Invitrogen). Hybridizations were performed in humidity chambers (Corning, Corning, NY, USA) at 65°C for 16 hours. Slides were then washed, dried, and scanned using an Axon GenePix 4000 scanner (Axon)

### cDNA microarray data analysis

Microarray images were quantified using GenePix 4.0 software (Axon). Data were uploaded to the Longhorn Array Database as well as the Acuity 4.0 database (Axon). Array normalization and spot filtering were done using Acuity. We excluded spots with sum of median intensity less than 150 or spots that had been flagged during visual gridding. For subsequent analysis, we used the log_2 _of the background subtracted, normalized median spot intensities of ratios from the two channels (Cy5/Cy3). Data were normalized in Acuity such that the mean of the median of ratios of all good features equaled 1.0. Data for each time course were then transformed to show expression changes for each gene relative to the zero time point, by dividing the median of ratios in the time zero replicates from the corresponding data measured in each time point. Three replicate time-courses were done for each treatment and only genes with at least 80% of expression measurements present were considered technically adequate and were included in the subsequent analysis.

To identify consistently induced genes, we selected those with more than twofold change (median of ratios) in at least 15 array experiments, which yielded 1,552 cDNA probes. After removing cDNAs with no known Unigene annotation or those that matched multiple Unigene clusters, we were left with 1,304 cDNAs that were subjected to hierarchical clustering. By using average linkage clustering, two main clusters of genes showing consistent induction (after removing two small clusters of inconsistently varying genes) were picked for further analysis, which contain 237 genes represented by 278 cDNA probes. Consistently repressed genes whose expression was reduced relative to the time zero samples in at least 85% of the arrays were selected directly from the expression data. This set with 237 genes represented by 250 cDNA probes was then hierarchically clustered.

To identify genes differentially expressed between different treatment groups (Class II and Class III genes), a Student's *t*-test was performed and the FDR was calculated using the Benjamini-Hochberg method in Acuity. For Class II genes, the two groups were all the serum treated samples as one group, and all the GF treated samples as another. For Class III genes, the two groups were all the foreskin fibroblast samples as one group and all the lung fibroblast samples as another. Genes with FDR = 0.01 were considered expressed differently between two groups. Hierarchical clustering was also done in Acuity or by using Cluster and TreeView. Clones were queried in the SOURCE database [59]. Unigene ID, gene symbol, and GO annotation were thus obtained and used for functional classification.

### miRNA microarrays and analysis

DNA oligonucleotides corresponding to 206 miRNAs and controls were purchased from Ambion. Sequences of these miRNAs are provided in Additional data file 9. These probes were resuspended in 3x saline sodium citrate (SSC) with 0.01% SDS at 50 μM and spotted on Nexterion epoxy Slide E (Schott, Mainz, Germany) Slides were rehydrated and dried normally, and were then blocked in a solution containing 100 mM ethanolamine, 1 M Tris (pH 9.0), and 0.1% SDS for 20 minutes at 50°C, then rinsed with water several times and spun dry. Labeled miRNAs were mixed with 3x miRNA hybridization buffer (Ambion) to 36 μl and heated at 95°C for 3 minutes. Slides were hybridized at 42°C for 14 to 16 hours and scanned like the cDNA arrays described above. Image gridding and preliminary analysis were done in GenePix Pro 5.0 (Axon). Data were then imported into Acuity 4.0 (Axon) for further normalization and analysis. Normalization was done by a ratio-based method. Generally, spots with intensity lower than 150 or with median of ratios higher than 5 or lower than 0.2 were excluded from the normalization. The ratio for each spot was adjusted so that the overall mean of medians of ratios equaled 1.0. Only those spots with intensity higher than 150 were selected for further analysis. Hierarchical clustering was done using complete linkage with uncentered Pearson correlation.

### Western blot analysis

Quiescent 2091 and WI-38 cells were treated for 2 hours with medium containing 10% FBS or 50 ng/ml EGF, 100 ng/ml FGF and 40 ng/ml PDGF. Cell lysates (50 mg of protein) were run on a 4% to 15% polyacrylamide gel (BioRad, Hercules, CA, USA) and transferred to PVDF membrane (Millipore, Billerica, MA, USA). The membrane was then blotted with the indicated antibody and processed using chemiluminescence (Santa Cruz, Santa Cruz, CA, USA). Antibodies used in this experiment were anti-*EGFR *(sc-03), anti-*H-Ras *(sc-520) and anti-*ERBB2 *(sc-7301), all from Santa Cruz Antibody. All western blot experiments were performed at least three times.

## Additional data files

The following additional data are available with the online version of this paper. Additional data file 1 lists the expression data of Class I genes. Additional data file 2 shows the DAVID analysis of enriched GO categories within Class I, Class II and Class III genes. Additional data file 3 is a histogram of expression peak timing. The graph shows the number of genes with peak expression levels at each of the indicated times after stimulation of quiescent cells, for each of the four treatments. We calculated the peak time for each Class I gene (genes induced by all treatments) for each time course. Data for skin and lung fibroblasts were considered in combination. Since we had two cell lines and three biological repeats of each time course, for each GF treatment, we took the median time point at which a gene showed its peak expression level. We then calculated the percentage of genes peaking at each time point for each treatment. EGF, FGF and PDGF tend to induce their peak expression levels between 2 and 4 hours, but expression peaks in response to serum are delayed up to 8 hours. Additional data file 4 shows the dose response of skin fibroblasts to FGF. Skin fibroblasts were treated with the indicated concentrations of FGF for 2 hours. Higher concentrations resulted in higher expression levels of induced genes, comparable to the expression levels seen after treatment of the same cells with serum. Data have been normalized to the time zero sample value. However, although serum treatment resulted in overall higher expression levels, their peaks of expression were generally delayed as compared to FGF treatment. Additional data file 5 lists the expression data of Class II genes. Additional data file 6 shows the tissue-specific gene expression in skin and lung fibroblasts. Relative gene expression levels of quiescent skin fibroblasts and lung fibroblasts are shown such that red indicates high expression in skin fibroblasts and green indicates high expression in lung fibroblasts. The right hand portion of the cluster shows data from this study. Since a common universal reference sample was used in all experiments, we divided the ratio of skin versus reference by the ratio of lung versus reference to obtain skin versus lung data sets from a number of independent replicate experiments. The left hand side shows comparative data from an earlier study [6]. Additional data file 7 lists the expression data of Class III genes. Additional data file 8 lists the expression data of miRNA genes. Additional data file 9 provides the sequences of all miRNAs included on the miRNA microarray.

## Supplementary Material

Additional File 1 Expression data of Class I genes Click here for file

Additional File 2 DAVID analysis of enriched GO categories within Class I, Class II and Class III genes Click here for file

Additional File 3 The graph shows the number of genes with peak expression levels at each of the indicated times after stimulation of quiescent cells, for each of the four treatments. We calculated the peak time for each Class I gene (genes induced by all treatments) for each time course. Data for skin and lung fibroblasts were considered in combination. Since we had two cell lines and three biological repeats of each time course, for each GF treatment, we took the median time point at which a gene showed its peak expression level. We then calculated the percentage of genes peaking at each time point for each treatment. EGF, FGF and PDGF tend to induce their peak expression levels between 2 and 4 hours, but expression peaks in response to serum are delayed up to 8 hours. Click here for file

Additional File 4 Skin fibroblasts were treated with the indicated concentrations of FGF for 2 hours. Higher concentrations resulted in higher expression levels of induced genes, comparable to the expression levels seen after treatment of the same cells with serum. Data have been normalized to the time zero sample value. However, although serum treatment resulted in overall higher expression levels, their peaks of expression were generally delayed as compared to FGF treatment Click here for file

Additional File 5 Expression data of Class II genes Click here for file

Additional File 6 Relative gene expression levels of quiescent skin fibroblasts and lung fibroblasts are shown such that red indicates high expression in skin fibroblasts and green indicates high expression in lung fibroblasts. The right hand portion of the cluster shows data from this study. Since a common universal reference sample was used in all experiments, we divided the ratio of skin versus reference by the ratio of lung versus reference to obtain skin versus lung data sets from a number of independent replicate experiments. The left hand side shows comparative data from an earlier study [6] Click here for file

Additional File 7 Expression data of Class III genes Click here for file

Additional File 8 Expression data of miRNA genes Click here for file

Additional File 9 Sequences of all miRNAs included on the miRNA microarray Click here for file
