# Supplementary figures and images for: PI3K signaling and miRNA expression during the response of quiescent human fibroblasts to distinct proliferative stimuli
Source: Genome Biol. 2006 May 31;7(5):R42. doi: 10.1186/gb-2006-7-5-r42 (PMC1779520; doi:10.1186/gb-2006-7-5-r42)

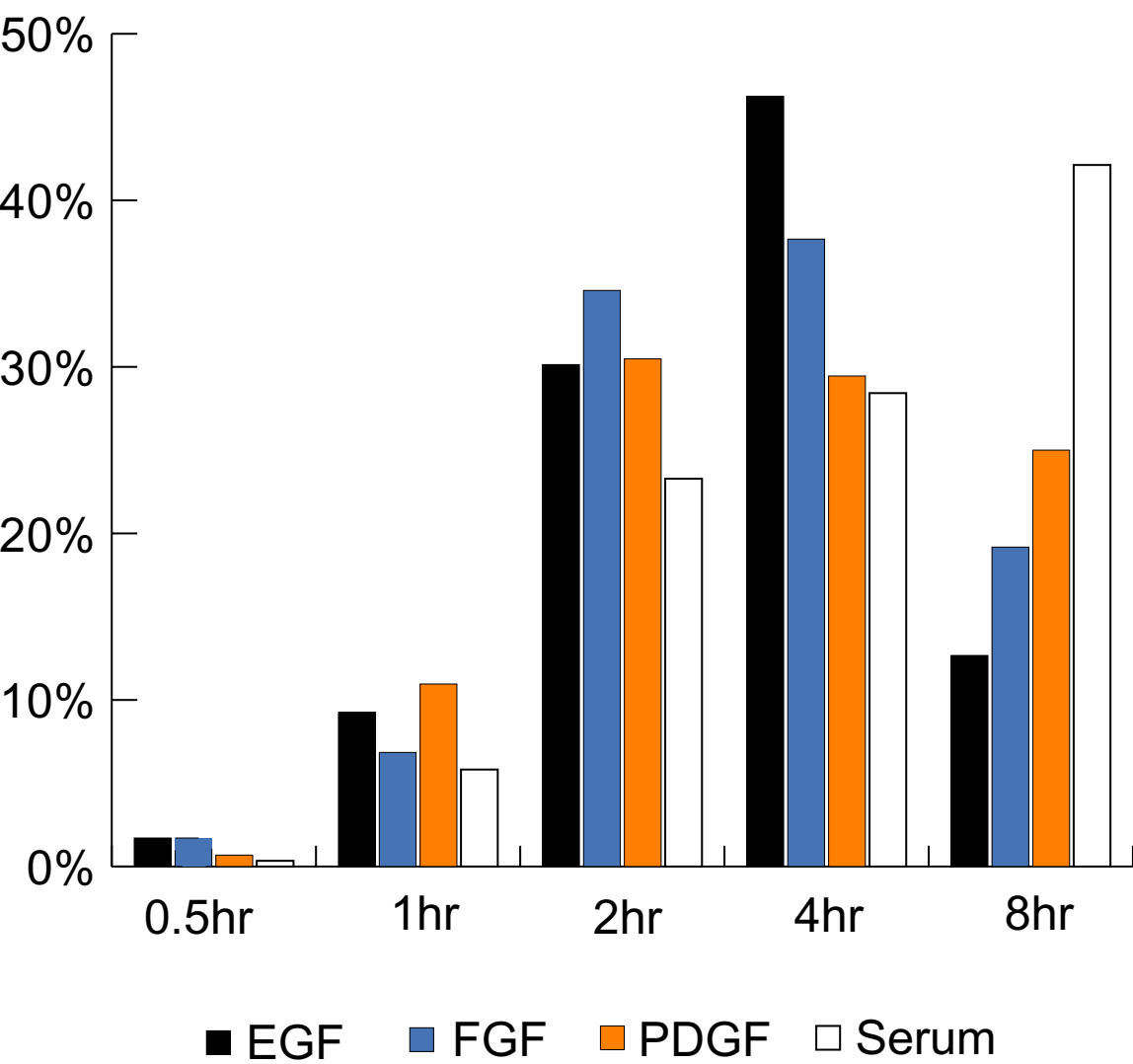

Supplement: Additional File 3 — The graph shows the number of genes with peak expression levels at each of the indicated times after stimulation of quiescent cells, for each of the four treatments. We calculated the peak time for each Class I gene (genes induced by all treatments) for each time course. Data for skin and lung fibroblasts were considered in combination. Since we had two cell lines and three biological repeats of each time course, for each GF treatment, we took the median time point at which a gene showed its peak expression level. We then calculated the percentage of genes peaking at each time point for each treatment. EGF, FGF and PDGF tend to induce their peak expression levels between 2 and 4 hours, but expression peaks in response to serum are delayed up to 8 hours. [file gb-2006-7-5-r42-S3.pdf]

FGF 5 ng/ml

FGF 25 ng/ml

FGF 50 ng/ml

FGF 75 ng/ml

FGF 135 ng/ml

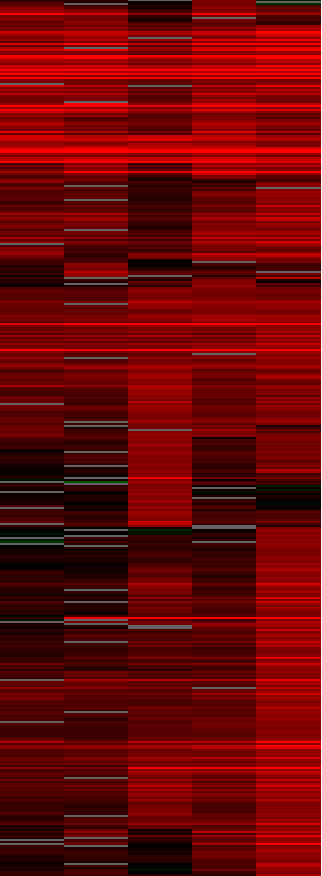

Supplement: Additional File 4 — Skin fibroblasts were treated with the indicated concentrations of FGF for 2 hours. Higher concentrations resulted in higher expression levels of induced genes, comparable to the expression levels seen after treatment of the same cells with serum. Data have been normalized to the time zero sample value. However, although serum treatment resulted in overall higher expression levels, their peaks of expression were generally delayed as compared to FGF treatment [file gb-2006-7-5-r42-S4.pdf]
