# Supplementary material for: PI3K signaling and miRNA expression during the response of quiescent human fibroblasts to distinct proliferative stimuli
Source: Genome Biol. 2006 May 31;7(5):R42. doi: 10.1186/gb-2006-7-5-r42 (PMC1779520; doi:10.1186/gb-2006-7-5-r42)
Supplement: Additional File 6 — Relative gene expression levels of quiescent skin fibroblasts and lung fibroblasts are shown such that red indicates high expression in skin fibroblasts and green indicates high expression in lung fibroblasts. The right hand portion of the cluster shows data from this study. Since a common universal reference sample was used in all experiments, we divided the ratio of skin versus reference by the ratio of lung versus reference to obtain skin versus lung data sets from a number of independent replicate experiments. The left hand side shows comparative data from an earlier study [6] [file gb-2006-7-5-r42-S6.pdf]

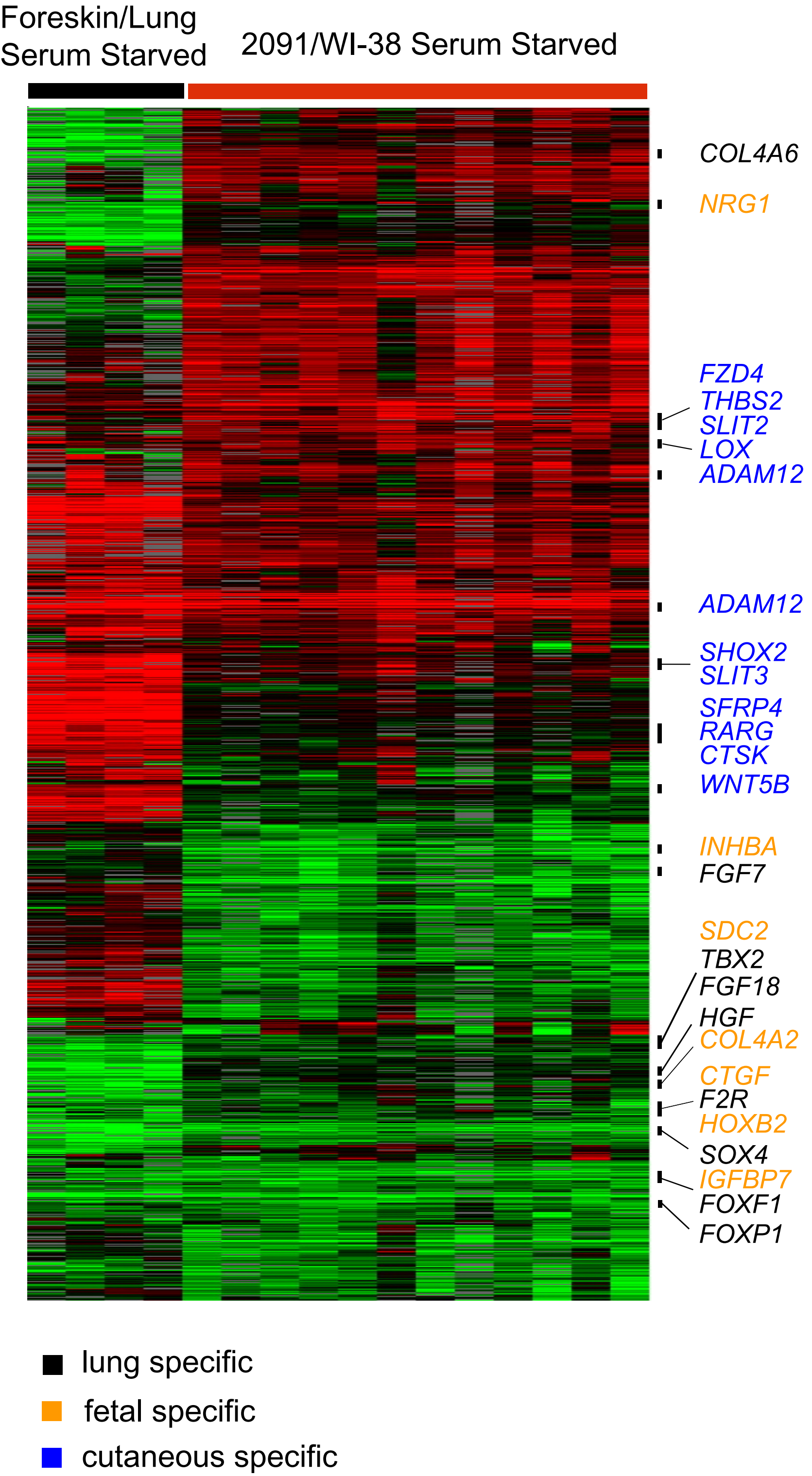
Foreskin/Lung
Serum Starved
2091/WI-38 Serum Starved
COL4A6
NRG1
FZD4
THBS2
SLIT2
LOX
ADAM12
ADAM12
SHOX2
SLIT3
SFRP4
RARG
CTSK
WNT5B
INHBA
FGF7
SDC2
TBX2
FGF18
HGF
COL4A2
CTGF
F2R
HOXB2
SOX4
IGFBP7
FOXF1
FOXP1
lung specific
fetal specific
cutaneous specific
